# Welfare Reform and Substance Abuse Treatment for Welfare Recipients

**Published:** 2006

**Authors:** Jon Morgenstern, Kimberly A. Blanchard

**Affiliations:** Jon Morgenstern, Ph.D., is vice president and director of the Division of Health and Treatment Research and Analysis, and Kimberly A. Blanchard, Ph.D., is a research associate, both at the National Center on Addiction and Substance Abuse (CASA), Columbia University, New York, New York

The 1996 welfare reform law replaced the existing entitlement to cash welfare with a transitional program of temporary aid that has employment as its primary goal. Among the key provisions of the welfare reform legislation were mandatory time limits on benefits and work requirements for all recipients, including those with substance use disorders.

Changes brought about by the welfare reform law have important implications for the organization of substance abuse services and the well-being of disadvantaged children whose parents have substance use disorders. The overall effect of welfare reform could be positive. It gives States wide latitude to design programs to help low-income people attain self-sufficiency and has increased interest in developing innovative programming for hard-to-employ welfare populations, including those with substance use disorders (Berlin 2001). This interest could lead to increased funding for treatment, new services such as screening, better integration of needed ancillary services (i.e., medical care, child care, help with housing and transportation), and more accountability for outcomes on the part of programs and government systems. Conversely, welfare reform may have a profoundly negative impact on low-income people with substance use disorders and the programs that serve them. The policy of sanctioning welfare recipients for failure to comply with new welfare regulations and a punitive atmosphere at welfare offices may drastically reduce the number of low-income Americans with substance use disorders who receive public aid, and treatment programs dependent on public funds may face a resulting loss of revenue.

Research on substance use disorders in the context of welfare reform has primarily focused on four questions:

1. What is the prevalence of substance use and substance use disorders among Temporary Assistance to Needy Families (TANF) recipients?
2. To what extent are substance use disorders and co-occurring problems a barrier to employability?
3. Can screening strategies in welfare settings successfully identify and refer clients to substance abuse treatment?
4. What types of services do these clients need to attain self-sufficiency?

This article reviews findings on these questions and offers suggestions for how these findings can inform policy and future research.

## Prevalence of Substance Abuse in Welfare Populations

Prevalence rates for substance use problems among TANF recipients vary widely depending on study methodology and on how problem use is defined. Most recent studies cite survey data that relies exclusively on administrative data or self-reports of substance use (Jayakody et al. 2000), both of which are likely to underestimate the true prevalence of substance use disorders (Metsch and Pollack 2005). Data from the 1998 National Household Survey on Drug Abuse (NHSDA) indicate that in the previous year 7.5 percent of TANF recipients were alcohol dependent and 4.5 percent were dependent on illicit drugs (Pollack et al. 2002). According to other studies using self-report data, 6 to 10 percent of TANF recipients were dependent on either alcohol or other drugs (Jayakody et al. 2000; Schmidt et al. 1998; Chandler and Meisel 2000; Grant and Dawson 1996). It is more difficult to determine the prevalence of problem users who are not dependent. Overall prevalence data obtained from numerous waves of the NHSDA and the more recent National Survey on Drug Use and Health (NSDUH) suggest that illicit substance use was about twice as common among female welfare recipients as among other women with dependent children who did not receive public assistance (Jayakody et al. 2000; Pollack et al. 2002). Data from the 2002 NSDUH show that about 22 percent of female welfare recipients used illicit drugs at least once in the year before the survey (Pollack et al. 2002).

The exclusive reliance on self-report data is a serious limitation of these findings. Many experts now consider data on the prevalence of substance use drawn from the NHSDA to be unreliable because of underreporting. Findings from a study comparing self-report and biological measures of substance use among welfare recipients suggest that substantial underreporting may occur. Kline and colleagues (1998) surveyed substance use among a representative sample of welfare recipients in New Jersey. They found that 12 percent self-reported cocaine use, but 25 percent tested positive for cocaine use based on hair sample analyses. Overall, prevalence rates from surveys are the best estimates available but should be seen as the lower bounds of the problem.

## Co-Occurring Problems and Barriers to Employment

Although substance use disorders affect a minority of TANF recipients, findings from many studies have shown that TANF recipients who use substances have high rates of other co-occurring problems (Chandler and Meisel 2000). For example, Pollack and colleagues (2002) found that twice as many women who reported illicit drug use met criteria for a psychiatric disorder compared with women who did not use drugs. Studies examining women with substance use disorders in welfare settings have found that these women had chronic substance use problems and experienced many other barriers to employment. For example, one study (Morgenstern et al. 2003*a*) examined the barriers to employment faced by women who screened positive for substance abuse in welfare offices in comparison with a sample of non-substance-abusing TANF women. On average, compared with non-substance-abusing women, more than twice as many substance-abusing women experienced severe barriers to employment, such as psychiatric problems, housing problems, legal problems, and domestic violence (see the accompanying figure). Research indicates that the presence of these barriers and especially the co-occurrence of multiple barriers are associated with lower likelihood of employment (Pollack et al. 2002).

Studies which have followed TANF clients over time consistently have found that having a substance use disorder predicts poorer employment outcomes (Danziger et al. 2000). For example, a longitudinal study in two California counties found that the rates of employment among TANF recipients without substance abuse were about double those of recipients with substance abuse (Chandler and Meisel 2002).

## Screening and Identification of Substance Use Problems in Welfare Settings

Most people are reluctant to disclose having a substance use problem because of the stigma involved (Metsch and Pollack 2005). TANF recipients are especially reluctant because of added concerns about losing their welfare benefits. A number of welfare systems attempt to screen for substance use problems using generic screening methods (Morgenstern et al. 2001*a*), in which caseworkers conduct the screening, all recipients are screened at the point of benefit determination, and short paper-and-pencil self-report surveys are used. Generic screening methods have yielded modest results, with States reporting 1 to 4 percent positive response rates (Morgenstern 1999). Thus, this type of screening is not as successful at identifying substance use problems among TANF clients as originally hoped.

In a more recent study (Morgenstern et al. 2001*a*), researchers implemented a specialized screening method in which high-risk populations (TANF recipients most likely to have a substance use problem) received intensive screening conducted by specially trained staff, and interview methods were used to establish rapport and facilitate self-disclosure. Specialized screening methods yielded much higher rates of identification (10 to 49 percent) than did generic screening methods (Morgenstern et al. 2001*a*). Overall, States need considerable help to improve their strategies for identifying substance use problems, and specialized screening strategies are significantly better than simply relying on self-report questionnaires.

## Specialized Interventions

Little research to date has examined interventions designed specifically to address substance use problems in women on welfare. Generally, studies indicate that these women have myriad co-occurring problems in the areas of mental health, domestic violence, and medical care, as well as legal issues. Thus, interventions designed to specifically address substance abuse may not effectively address the significant and chronic problems experienced by women receiving welfare benefits. Interventions that provide gender-specific services and coordination across multiple service domains to address the co-occurring problems these women experience may be most effective.

Two recent studies examined the effectiveness of case management (CM) at addressing the multiple problems experienced by substance-abusing women on welfare: CASAWORKS for Families (CWF) (Morgenstern et al. 2003*b*) and CASASARD (Morgenstern et al. 2001*b*). These CM interventions, designed specifically for TANF women, provided linkages to needed wraparound services in many areas, including housing assistance, mental health treatment, medical treatment, child care, and transportation. Additionally, when possible, services were tailored to women by referring clients to treatment programs that had female therapists, women-only groups, and child care.

**Figure:**
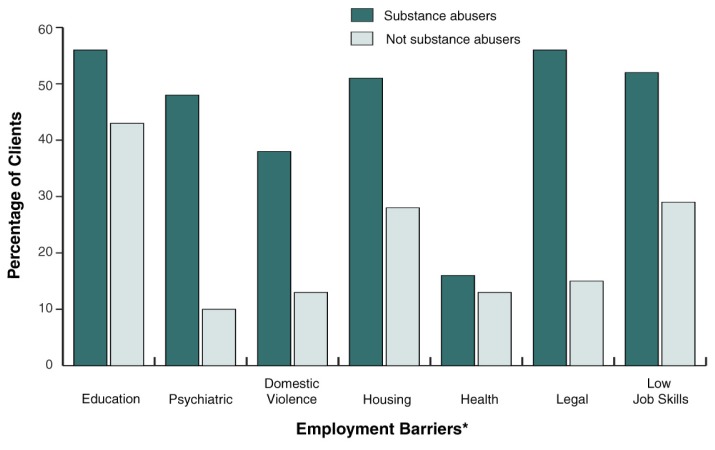
Prevalence of barriers to employment among substance-abusing and non-substance-abusing female welfare recipients. On average, more than twice as many substance-abusing women experienced severe barriers to employment compared with non-substance-abusing women. ^*^Definitions: Education: Less than a high school education Psychiatric: Diagnosis of post-traumatic stress disorder or severe depressive symptoms Domestic violence: Severe physical domestic violence Housing: Unstable or temporary housing Health: Fair or poor health and scoring in the lowest quartile on health when compared with a national sample Legal: Ever arrested Low job skills: Little or no job skills SOURCE: Morgenstern et al. 2003*a*.

CWF was a demonstration program testing an intensive intervention for TANF women with substance use problems in 10 counties around the country (Morgenstern et al. 2003*b*). CWF offered client-level case management and fostered interagency coordination to ensure that clients had access to ancillary services. The study did not employ a control group, but researchers conducted a rigorous evaluation of CWF with 698 women receiving treatment at 10 sites. An independent evaluation of this demonstration project produced promising findings (McLellan et al. 2003). Women had high rates of retention (51 percent were still in treatment 6 months after beginning treatment) and received substantial amounts of ancillary services. Followup at 12 months showed that the women had significant and meaningful reductions in substance use (78 percent reported no heavy alcohol use in the previous 6 months), increases in employment (41 percent were employed at least part-time at the 12-month followup), and decreases in welfare dependency.

Although this was not a randomized clinical trial, results indicate that integrated care programs may be effective for addressing the multiple needs of substance-abusing women on welfare. However, such programs require careful attention to the coordination of services across agencies as well as other implementation issues.

CASASARD was the result of a collaboration between researchers and the New Jersey Department of Human Services (NJDHS) to compare intensive case management (ICM) with usual care (UC) in two counties in New Jersey (Morgenstern et al. 2001*b*). Women meeting criteria for substance dependence were identified in welfare offices and randomly assigned to receive either ICM or UC. ICM was designed to assist in engaging and retaining clients in care, assessing and linking clients to ancillary services including employment training, and providing long-term monitoring and continuity of care. In addition, ICM clients received incentives for attending substance abuse treatment. A total of 302 clients were randomly assigned to receive ICM or UC, and 96 percent of clients were followed for 15 months after intake. Results are promising, indicating that ICM more than doubled the rates of engagement and retention in substance abuse treatment (42 percent versus 18 percent), and nearly twice as many ICM clients as UC clients were completely abstinent at 15 months (43 percent versus 26 percent) (Morgenstern et al. 2002). However, the ICM clients showed no differences in employment outcomes at 15 months in comparison with women who received standard care (Morgenstern et al. 2002). CASASARD was a well-designed, well-implemented, rigorously evaluated, randomized clinical trial, and although findings suggest that ICM can improve outcomes for these difficult-to-treat women, implementation of the full model, especially employment training, is critical to success. Currently, NJDHS has implemented ICM in six New Jersey counties based on results of this trial.

## Summary and Implications for Policy and Research

Overall, best estimates suggest that 8 to 20 percent of women on TANF have a substance use problem that probably interferes with their functioning (Metsch and Pollack 2005). Women with substance use disorders experience substantially more barriers to employment and are less likely to become employed and more likely to be sanctioned and lose welfare benefits. Screening procedures are being used in many States to identify TANF recipients with substance use disorders, but findings to date suggest that screening identifies only a minority of those with substance use disorders. Evidence from a rigorous random assignment study indicates that more intensive interventions can yield better treatment engagement rates and substance use outcomes.

Overall, available research supports policies that have identified TANF recipients with substance use disorders as part of a group of hard-to-employ (HtE) recipients who require more intensive services than the typical brief training programs followed by work assignments that most welfare settings offer. However, substantially more research is needed to provide critical answers to the questions posed at the beginning of this article regarding an evaluation of the broad impact of welfare reform on disadvantaged mothers with substance use problems. Given the funding constraints and particular challenges associated with conducting this type of research, it is critical to find new ways for substance abuse health services researchers to collaborate with State and Federal welfare agencies on this agenda. Overall, Federal and State governments face important questions regarding the handling of HtE welfare recipients: What services are effective in helping HtE recipients move toward self-sufficiency? Can States afford to provide these services? And what are the consequences, both human and economic, when such families are removed from the welfare roles? Further research is urgently needed to inform policy in this area.

## References

[b1-63-67] Berlin G (2001). The 30-year tug-of-war: Can reform resolve welfare policy’s thorniest conundrum?. Brookings Review.

[b2-63-67] Chandler D, Meisel J (2000). The Prevalence of Mental Health, Alcohol and Other Drug, and Domestic Violence Issues Among CalWORKS Participants in Kern and Stanislaus Counties.

[b3-63-67] Chandler D, Meisel J (2002). Alcohol and Other Drug, Mental Health, and Domestic Violence Issues: Effects on Employment and Welfare Tenure After One Year.

[b4-63-67] Danziger S, Corcoran M, Danziger S (2000). Barriers to Employment of Welfare Recipients.

[b5-63-67] Grant BF, Dawson DA (1996). Alcohol and drug use, abuse, and dependence among welfare recipients. American Journal of Public Health.

[b6-63-67] Jayakody R, Danziger S, Pollack H (2000). Welfare reform, substance use and mental health. Journal of Health Politics, Policy and Law.

[b7-63-67] Kline A, Bruzios C, Rodriguez G, Mammo A (1998). Substance Abuse Needs Assessment Survey of Recipients of Temporary Assistance for Needy Families (TANF).

[b8-63-67] McLellan AT, Gutman M, Lynch K (2003). One-year outcomes from the CASAWORKS for Families intervention for substance-abusing women on welfare. Evaluation Review.

[b9-63-67] Metsch LR, Pollack HA (2005). Welfare reform and substance abuse. Milbank Quarterly.

[b10-63-67] Morgenstern J (1999). Why Are Screening and Treatment Referral Rates Lower Than Expected in the New Jersey Substance Abuse Initiative?.

[b11-63-67] Morgenstern J, Riordan A, DePhilippis D (2001a). Specialized Screening Approaches Can Substantially Increase the Identification of Substance Abuse Problems Among Welfare Recipients.

[b12-63-67] Morgenstern J, Riordan A, McCrady BS (2001b). Barriers to Employability Among Women on TANF With a Substance Abuse Problem.

[b13-63-67] Morgenstern J, Riordan A, McCrady BS (2002). Intensive Case Management Improves Welfare Clients’ Rates of Entry and Retention in Substance Abuse Treatment.

[b14-63-67] Morgenstern J, McCrady BS, Blanchard KA (2003a). Barriers to employability among substance dependent and nonsubstance-affected women on federal welfare: Implications for program design. Journal of Studies on Alcohol.

[b15-63-67] Morgenstern J, Nakashian M, Woolis DD (2003b). CASAWORKS for Families: A new treatment model for substance-abusing parenting women on welfare. Evaluation Review.

[b16-63-67] Pollack HA, Danziger S, Seefeldt K, Jayakody R (2002). Substance use among welfare recipients: Trends and policy responses. Social Service Review.

[b17-63-67] Schmidt L, Weisner C, Wiley J (1998). Substance abuse and the course of welfare dependency. American Journal of Public Health.

